# A Systematic Review of Data Collection Techniques Used to Measure Preschool Children’s Knowledge of and Preference for Physical Activity

**DOI:** 10.3390/ijerph16060964

**Published:** 2019-03-18

**Authors:** Nicola Wiseman, Christin Rossmann, Neil Harris

**Affiliations:** 1 School of Medicine, Griffith University, Queensland, 4215, Australia; n.harris@griffith.edu.au; 2 Department of Health Sciences, University of Applied Sciences, Hamburg, 21033, Germany; c.rossmann58@gmail.com

**Keywords:** physical activity, preference, knowledge, child-preschool, evaluation

## Abstract

Background: Early childhood has been identified as a crucial period in which children develop physical activity preferences and behaviors. Both the knowledge of and preferences for physical activity are key proximal indicators of activity choices in children. Thus, accurate data collection tools are required to measure these variables. This review evaluates the data collection techniques that have been utilised to assess preschool children’s knowledge of and preference for physical activity, and examines the validity and reliability of existing techniques. Methods: A systematic search for relevant studies published from 1980 through to December 2017 was conducted via ProQuest, CINAHL, Embase, Scopus, ERIC, PubMed, MEDLINE, and ScienceDirect. Results: Fourteen studies were eligible for inclusion in the review. The identified studies employed a limited but disparate range of techniques to assess children’s physical activity knowledge and preferences. Findings reveal that four techniques were consistently used across the reviewed studies, including: interviews, structured play-based activities, questionnaires, and observations. Only four out of 14 included studies reported the assessment of the validity of the data collection tool used, and six reported testing the measures for at least one type of reliability. Conclusion: There is a need for validated and reliable measures to assess children’s knowledge of and preference for physical activity. Greater consideration is required to align data collection techniques with the characteristics, needs and abilities of this study population.

## 1. Background

Childhood overweight and obesity is an important public health concern, with over 41 million children being overweight globally [1]. Overweight and obesity can affect a child’s immediate physical and mental health, educational attainment, and quality of life [1,2,3]. Obese children are very likely to remain obese as adults, placing them at risk of chronic illness [1,4,5]. There are many factors that contribute to the rising prevalence of childhood overweight and obesity, with physical inactivity and screen time being key factors [6]. Early childhood has been identified as a crucial period for establishing positive activity behaviours, as this is a key age at which children develop preferences for physical activity (PA) and learn important motor skills [7,8,9,10]. Thus, promoting and encouraging PA within early childhood may facilitate a pattern of participation in PA across the lifespan.

Over the last decade, there has been a dramatic increase in interventions that seek to promote PA participation in young children [11]. These interventions often measure increases in PA through the use of heart rate monitors, motion sensors (accelerometers, pedometers) and indirect measurements, including self-report measures, interviews, proxy-reports, and diaries [11,12,13,14,15]. The effectiveness of PA interventions targeting overweight and obesity has often been evaluated through measures of weight, body mass index (BMI), BMI *z*-scores and waist circumference [11,12,13,14,15]. Emerging research has begun to incorporate the measurement of preschool children’s lifestyle knowledge and preferences as proximal outcomes that shape PA [16].

Both knowledge of and preferences for PA have been linked to PA choices in preschool children [7,17]. The first five years of life is a key period in which children are accumulating knowledge and developing skills that can shape health behaviors [8,10]. Several studies have revealed that children as young as three years old have the ability to identify activities that make their body healthy [7,18,19]. Children who are able to identify healthy activities may be more likely to apply this knowledge, and to select activities that promote their body’s health [20]. As preschool children grow older and gain autonomy, children operationalise these understandings into health behaviours [21]. Furthermore, given the potential ongoing influence of children’s knowledge of physical activity throughout their lives, it is important to identify knowledge gaps or misperceptions that may exist at this age, and to establish effective ways to improve this knowledge and understanding [7,18,19]. While knowledge alone is not a strong predictor of health behaviours, understanding children’s knowledge of health-related behaviours is important, as it is easier to establish positive health attitudes than to alter negative health behaviour and attitudes in later life [21].

PA factors such as the age, gender, ethnicity and weight status of a child, and parental factors such as parental modelling and activity preferences, may shape children’s activity preferences [22,23]. Beyond these factors, children are more likely to participate in physical activities for the reasons of fun and enjoyment, if they are ‘good’ at the activity or if a friend participates [24]. Parents’ report that facilitating regular PA for their children is most difficult with children who prefer more sedentary activities [25]. Measuring a child’s preferences for PA can therefore be a useful means to develop targeted and effective lifestyle interventions, determine the impact of such interventions, and to understand why children participate in certain activities. Given the diverse range of factors shaping a child’s PA preferences, a child’s account of their own PA preferences might differ from those reported by their parents. Thus, working with children to obtain self-reported activity preferences is important [26]. 

The assessment of a child’s PA knowledge and preferences is gaining recognition as an important means for measure the proximal impacts of PA interventions that are targeted at this age group. As such, as more techniques to assess these variables become available, there is a need to ensure that these measures are robust and reliable [20,21]. This paper will examine the techniques that have been used to measure 3- to 5-year-olds’ PA knowledge and preferences, by reviewing the validity and reliability of these techniques, and how they are supported by literature [27]. This review will be timely, in that it will offer educators and public health professionals a useful starting point for the development, selection, or improvement of techniques to evaluate PA interventions.

## 2. Methods

This review was conducted in accordance with the Preferred Reported Items for Systematic Reviews and Meta-Analyses (PRISMA) statement checklist [28]. For the purpose of the review, PA knowledge was defined as the ability to distinguish between sedentary and active activities, and to identify the need to be physically active, to promote health [7,10,24]. Physical activity preference was defined as being a child’s likes and dislikes of organised and unorganised activities, and was also related to relative liking of one type of activity more than the other [24,25].

### 2.1. Search Strategy

Articles were sourced from eight online databases, including: ProQuest, CINAHL, Embase, Scopus, ERIC, PubMed, MEDLINE and ScienceDirect. The search was conducted during the first week of December, 2017. Applying the Problem, intervention, comparison, outcome framework (PICO), a search strategy was devised using the following key words to identify publications for inclusion: (P) preschool* OR kindergarten OR early childhood OR child* AND (I) physical activity OR exercise OR sport AND (C) data collection OR technique, measurement OR assessment, OR evaluation AND (O) knowledge OR understanding OR preference*. The search was limited to articles in the English language, and published between 1980 and 2017. Titles and abstracts were scanned for relevance by one author [CR]. Reference lists of selected articles were also reviewed.

### 2.2. Study Selection

**Figure 1:**
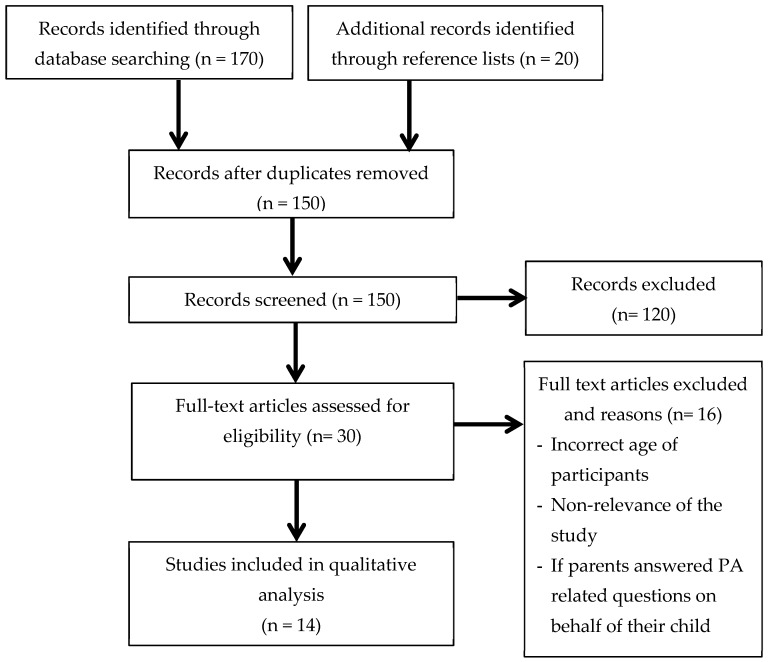
PRISMA flow chart depicting the article filtering process that was undertaken as part of the systematic review.

The PRISMA flow diagram (presented in Figure 1) shows the systematic literature search and selection processes that were followed by the authors. The selected studies were transferred to EndNote (version X8) and were assessed by two reviewers (NW and CR) to determine whether they met the inclusion criteria. This enabled authors to identify any disagreements relating to study suitability. During this process, articles that did not align with the inclusion criteria were removed, and the reason for exclusion was recorded. 

The following inclusion criteria were used to assess the relevance of each study: (1) assessment of preschool children’s knowledge and/or preferences of PA; (2) the technique used to assess each of the variables was explained in detail; (3) the paper was published in English; (4) participants included children between the ages of three and five years. When the ages of the participants was outside of the target age-range, the study was included if the results differentiated the 3–5 year old age group from other age groups. In cases where a study replicated a data collection technique developed in a previous study, the original article was sourced and included instead.

### 2.3. Data Extraction and Assessment of Methodological Quality

Information that was extracted from selected studies included: the study design, assessment method, sample size, age of the participants, description of the instrument, data regarding the tool validity, reliability, and pilot testing when available. Two independent researchers (NW and CR) assessed the quality of the selected papers, using the “Quality Assessment of Before-After (Pre-Post) Studies With No Control Group” from the National, Heart, Lung, and Blood Institute [29]. The tool includes 12 questions about the integrity and preciseness of information presented in the study, including: study design, sample size, description of methods, outcome measures, and statistical analysis; each study was classified accordingly as being good, fair or poor (Table 1). The quality assessment instrument was identified as the most appropriate, given the diversity of the study designs used in the included studies. 

## 3. Results

A total of 190 articles were initially identified from eight databases. After removing duplicates and adding articles identified from reference lists, 150 articles were screened for relevance. In total, 30 journal articles were retrieved for full review; of those, 14 articles met the inclusion criteria (see Figure 1). The most frequent reasons for the exclusion of articles were (1) non-relevance of the study (2) if parents answered the PA related questions on behalf of their child, and (3) the child’s age. 

Studies included in the review were published between 1985 and 2017, including 22 cross-sectional studies [6,7,18,20,23,30,33,34,35,36,37], one pre-post study [31], one quasi-experimental study [32] and one randomized controlled trial (RCT) [16]. The number of participants ranged from 17 to 1881 and included children aged three to 12 years. Of the reviewed articles, four studies assessed PA knowledge, five asked for PA preference and five studies tested for both PA knowledge and preference. The assessment of children’s knowledge and preferences was conducted one-on-one, in small groups with either two, three or five participants or in focus groups with 12 to 21 children (see Table 1). The time per assessment ranged from 10 to 30 min [33,34,35], though in most cases, this information was not reported. The quality ratings for the 14 included studies were mostly good (six studies) or fair (seven studies), with one study being rated as poor quality (see Table 1) [29].

### 3.1. Overview of Techniques

The reviewed studies utilised a limited but disparate range of techniques to measure preschool children’s PA knowledge and preferences. Three of the 14 studies incorporated qualitative research methods, seven used quantitative measurements, and four employed a mixed-method approach (Table 1). Techniques were assembled into four key types of method categories, including: (1) interviews, (2) structured-play-based activities, (3) questionnaires, and (4) observation. In seven of the 14 articles, a combination of two data collection techniques was used (Table 1).

#### 3.1.1. Interviews

As a technique, interviews can be defined as one-on-one or group interviews, incorporating the use of open-ended and closed questions [38]. Six studies used interviews to measure physical activity knowledge and preferences [7,18,23,33,34,37]. In most cases, interviews were conducted one-on-one [7,18,20,23], or with two to three participants [33]; only Darbyshire et al. [37] assessed PA knowledge and preferences in a focus-group. Of the reviewed studies, interviews took place in the child’s childcare centre [7,20,23], school [7,33,37], or home [18]. One study did not provide information about the setting of the interview [34].

Two studies [18,33] incorporated open-ended questions, for example “What is the best reason that exercising keeps us healthy?” [18] to assess PA knowledge, and one study used a combination of both open- and closed-ended questions to assess PA preferences [20]. Nguyen and others [34] used closed-ended questions to assess PA knowledge, for example, “To have a healthy body, would you ride a bike?” Three studies did not provide any examples of the questions asked [23,33,37]. 

#### 3.1.2. Structured Play-Based Activities

Seven studies used play-based activities, incorporating tasks such as role-play, drawings and photo collages. Structured play-based activities can be defined as task-centred activities in which the investigator uses set instructions to achieve a clear objective, such as a drawing or doll play [39]. Several studies incorporated craft/drawing activities as techniques to measure PA preference. For example, Bélanger and others [30] used photo collages, picturing active and sedentary activities. Children were asked to order the activities to reflect their preferences; for example, placing a green sticker for their first preference, a yellow sticker for their second and a red sticker for their third preferred activity. In the study by Cammisa et al. [20], children were asked to draw themselves in their preferred way of playing. This was followed by an interview in a quiet part of the classroom, asking questions on the basis of the drawing, such as, “What is your favourite game?” and “Why do you like it?”

Role play was used in three of the 14 reviewed studies [6,7,31]. In these studies, participants were asked to take care of a doll/bear, and to make decisions for the doll/bear. The caretaking role was used to ensure that the child’s choices were more likely about their knowledge of PA, rather than their preferences. These studies measured PA knowledge by asking children to point to the activity that makes the doll “healthy and grow big and strong” or that “helps the doll to stay healthy”. To determine PA preferences, children were asked to point to the activity that they liked best. Calfas and others [31] also asked the children further to rate their preferences on a scale from one to three; this was to assess the consistency of children’s responses. To measure PA knowledge, Lanigan [7] allowed for more freedom during the role-play activity. Children suggested leisure-time activities and responded to “less healthy requests” from the dolls, which included barriers to PA, such as bad weather, inability to master a skill, or preference for watching television. 

Darbyshire and colleagues [37] conducted focus group interviews with children, and incorporated activities to stimulate their thinking and responses regarding PA. At first, they used the ‘show me’ and ‘interested idiot’ approach, which have demonstrated efficacy in stimulating the thinking and responses of the children regarding PA. The ‘show me’ approach asked children to demonstrate activities and show where they participated in these activities. The ‘interested idiot’ approach involved the facilitator acting as an ‘idiot’ who had forgotten what play and activity was, like as a child, and who needed the help of the children to solve this problem. Second, to make focus groups more interactive, children were asked to draw and discuss a map of the social environments where they were most likely to participate in PA. This approach is called ‘mapping’. Third, to ensure that quieter children were engaged in the study, ‘photovoice’ was used to gather complementary data. Children were provided with disposable cameras, and asked to take photos over one week, and to write a brief comment for each photo they took, to explain what they wished to express with the photograph in relation to PA. For younger children, if necessary, the task was accomplished with an adult.

#### 3.1.3. Questionnaires

Of the 14 reviewed articles, six studies used a questionnaire to assess PA knowledge and preferences [6,16,31,32,35,36]. In all six studies, questions were read out loud to participants, and pictures were used to facilitate activities in which children were asked to sort a selection of photographs according to which activity they prefer (PA preference), or according to which activity would contribute positively towards health and which would not (PA knowledge). Children were asked to point or circle the picture of an activity, and were often presented with two pictures at a time, one showing children engaging in sedentary activities, such as playing with Lego [31] or active activities, such as climbing [6]. Calfas and others [31] was the only study to offer a detailed description of the photographs used, stating that the children represented in the photographs were five to six years old, and from several ethnic groups [36]. Further, Calfas et al. [31] described that when presenting children with photo pairs (one being sedentary and the other active), the activities that were displayed in both photos of the pair were of similar intensity and location: either outside or inside), solitary or group-based, and required the same amount of equipment. Two studies used electronic devices (computer or iPad) to present questionnaires, and to display images [6,36]. The assistance of an adult to read questions aloud, and to use the electronic devices, was required in all studies except one [33].

#### 3.1.4. Observation

Two studies used the observation of children’s activities as a tool to assess preferred activities [33,35]. Lopez-Dicastillo and others [33] observed children’s breaks and school meal times, everyday routine activities, school trips, and end-of-term activities [31]. Parrish et al., [35] observed children’s playground activities with the purpose of identifying common activities, to inform the development of a picture-selection questionnaire [33].

### 3.2. Validity and Reliability

Only four out of 14 of the included studies reported an assessment of the validity of the data collection tool used, and six reported testing measures for at least one type of reliability (see Table 1). Pilot-testing of the instrument was done in six studies [6,16,30,31,32,34].

#### 3.2.1. Physical Activity Knowledge

Two studies reported the assessment of the validity of their tools by measuring the extent of improvement in PA knowledge after a brief intervention. Calfas and colleagues [31] could not achieve significant results by measuring the extent of improvement in PA knowledge after a brief intervention, whereas a significant improvement was measured by Wiseman and others [6] (t = −3.40; *p* < 0.001). Céspedes et al. [16] tested their instrument for face and content validity, based on the opinions of a panel of experts in psychology, qualitative research, paediatrics, nutrition, child development and education [16]. In regards to reliability testing, three studies [6,34,36] assessed the test-retest reliability of their tool, resulting in low values, respectively (r = 0.43, *p* < 0.001; r = 0.47, *p* < 0.01; r = 0.58, *p* < 0.001). Céspedes and colleagues [16] attained a medium level for internal consistency (α = 0.64). Lanigan [7] tested for inter-rater reliability and findings, indicating 0.764 agreement [40].

#### 3.2.2. Physical Activity Preferences

To test the validity of their PA preference tool, Calfas and others [31] calculated the percentage agreement between the stated preferences and the actual choices of the activities. The results indicated that the overall percent agreement was at a chance level (i.e., 56.35%) [31]. Wiseman et al. [6] calculated the percentage agreement between the stated activity and the PA preferences at 63%, though this declined with age. Parrish and colleagues [35] assessed the validity of their tool using measures of convergent validity through the comparison of (1) an Actigraph Accelerometer Activity Data, and the Children’s Activity Questionnaire (CAP), and (2) observed playground activities and the CAP-data. The findings yielded non-significant correlations (see Table 1) [33]. The test-retest reliability of the tools for measuring PA preference were assessed in three studies [6,31,35], resulting in low values for correlation, respectively (r = 0.22, *p* = 0.03; r = 0.407, *p* < 0.0001; r = 0.03, *p* < 0.01). Wiseman et al. also tested for internal consistency (α = 0.53) [6].

## 4. Discussion

Physical activity knowledge and preferences are both key indicators of a child’s PA behaviour [7,17]. This systematic review reveals that, to date, few studies have assessed PA knowledge and preferences in preschool children. The primary goal of this review was to identify the tools that were used, to examine whether these techniques align with the specific needs of young children, including age-related developmental capabilities (short attention spans, limited verbal skills and lack of fine motor skills), and to make practical suggestions for the future measurement of preschool children’s PA knowledge and preferences. In most studies, researchers complemented their data collection techniques with additional materials, such as photographs/sketches, drawings or diaries, to stimulate the child’s responses. The literature underpinning the data collection techniques used in the reviewed studies, together with the reported testing for validity and reliability, will be used to consider the efficacy of assessment techniques.

### 4.1. Selecting Interview Questions

Six studies used interviews to measure PA knowledge and preferences. None of the reviewed studies that used an interview tested the authenticity of the measures, nor did they provide any information regarding pilot-testing. This represents a key limitation of the techniques used to date, as pilot testing is crucial for identifying misunderstandings resulting from miscommunications between the participating child and the researcher, problems with response sets or graphics, potential barriers, or other testing issues [41]. Nonetheless, pilot testing may have been conducted and not reported in the study.

Open-ended, closed-ended, and a combination of both question types were used. Some researchers suggest that open-ended questions in the absence of other verbal prompts or cues, particularly at the beginning of an interview, may be too challenging for children [42]. This could have been an issue in the study of Lasky and Eichelberger [18], who received a high percentage of no answers (40%) or nonsense answers to the question “What do you think is the best reason that exercising keeps you healthy?”. Due to limited cognitive and verbal capabilities of children, closed-ended questions at the beginning of an interview can help a child settle into the interview process [42]. However, using only closed-ended questions might neglect the child’s full experiences. It is more likely that the child is going to guess the “right” answer to please the adult or guardian [36,43]. Cammisa et al. [20] used both open- and closed-ended questions, and formulated short questions, such as, “What is your favourite game?” followed by, “Why do you like it?”. This method allows the child to become familiar with the topic, and to prepare for more complex questions [39]. Due to limited attention spans and linguistic processing capacities, easy-language, short questions, and one question at a time helps to reduce misunderstandings in the interview [44]. Giving additional prompts and asking direct questions at the beginning of the interview may help with engagement in the topic [42].

### 4.2. Using Structured-Play Based Activities

Play-based activities were used in six of the 14 reviewed studies, and included tasks such as role-play, drawings, ‘mapping’, ‘photovoice’, and ranking games. Drawing and craft activities served as engaging, task-centred data collection techniques to measure PA preferences. Cammisa et al. [20] asked participants to draw themselves in their preferred way of playing, followed by an interview that was guided by the drawing [20]. The use of drawings facilitates communication between the child and researcher as it can help to overcome the brevity of their verbal responses. Facilitating discussion with children allows for more elaborate responses that are more likely to reveal their knowledge and reflection of an event or concept, rather than their ability to repeat directives from caretakers. Further, a meta-analysis by Driessnack [45] provides strong evidence that the use of drawings is an appropriate and efficient method of facilitating communication with children [46]. Bélanger et al. [30] had participants rank their PA preferences onto a photo collage; although this is an age-appropriate method to assess PA preferences, several weaknesses to this technique were evident [30]. For example, the number of sedentary activities and active activities were uneven (eleven active vs three sedentary activities), so that this may have biased results due to the unequal representation of sedentary activities. Further, children’s responses may have been influenced by the responses of other children who were in the same group. A similar issue was also evident in the study conducted by Cammisa et al. [20], in which children within the same group reported similar PA preferences. Thus, although group activities can be a fun way to engage children in research, steps should be taken to minimise peer influences on responses [46].

Darbyshire and colleagues [37] used a ‘mapping’ technique, in which participants were asked to draw and discuss a map of the social environments where they were most likely to participate in PA [35]. This study also incorporated ‘photovoice’; this technique was used so that children who might be hesitant to contribute to a group discussion would feel more confident and autonomous if asked to take their own photographs. Limited information about the given instructions of the task, such as the number of photos, making it difficult to replicate the methodology. Nevertheless, the photographs were seen as a “valuable, visual complement to the focus group interviews with children” [37]. Thus, the use of photographs, in conjunction with a small description of what the child wants to express, is recommended [43]. Without the child’s interpretation, such analysis of this data would lack important insights, and remain adult-centric. Therefore, it is suggested that children should be included in the interpretation element when using this technique [37].

Role-play, in conjunction with the provision of a doll/bear, is a technique that has been developed to help children explain thoughts and behaviours [47]. In the reviewed studies, the use of bears/dolls appeared to be an effective way to measure PA knowledge [6,7,31]. Using dolls/bears allowed children to assume a caretaking role of the doll/bear, and encouraged them to select activities for the bear/doll to participate in, that would help the doll/bear “to grow big and strong and healthy”. Researchers who used this technique suggest that the ‘caretaker’ approach is an effective method for measuring children’s PA knowledge, because it enables the researcher to control for the influence of a child’s personal activity preferences [6]. However, the effectiveness of the use of dolls to elicit a caretaker response in children has not been adequately measured or tested, and it requires further research. Lanigan [7] also applied the caretaker approach, in which children suggested leisure-time activities, and responded to “less healthy requests” from the dolls. While this was based on empirical evidence (as reported by the study), no validity or reliability measurements have been conducted.

### 4.3. Questionnaires and the Selection of Materials and Prompts

Questionnaires were used extensively throughout the reviewed studies. All questionnaires included the use of pictures illustrating children engaging in a variety of activities. Pictorial prompts were used to create interactions between the researcher and the child, as a way of engaging children in the research. A researcher can work with young participants’ development of verbal abilities and shyness, by giving children the opportunity to communicate, through making a circle around the preferred answer, or through pointing. A key strength of pictorial prompts in a questionnaire is the short amount of time required, and the use of minimal or inexpensive materials. Thus, a large number of children can be assessed in field settings, to address a variety of research questions [6,31,39].

All six studies that used questionnaires as a technique incorporated pictures to assist the children’s ability to respond. However, it was evident from the reviewed studies that a number of factors need to be considered when selecting photographs to reduce bias in the measurement of preschool children’s PA knowledge and preferences. To assess appropriateness, five studies tested the questionnaires for face or content validity through expert reviews or pilot-testing [6,16,31,32,35]. Validity and pilot-testing allows researchers to identify any issues with the tool, including the appropriateness of the tool content, issues with delivery, or how the participants understand what is being asked of them. This information can assist in refining the tool before it is used for data collection. For example, to gain valid results regarding children’s PA knowledge and preferences, the child needs to be able to understand and recognise the active and sedentary activities that are presented in the pictures [48].

Familiarity is a key consideration when assessing children’s PA knowledge and their preferences. Children are less likely to select unfamiliar activities or activities that they do not have the motor skills to complete as preferred activities [31]. Further, when selecting physical activities for photographs it is important to consider the level of risk that they represent. Children may be more inclined to select sedentary behaviours, or the ‘safe option’ as health-promoting activities, biasing their scores of PA knowledge [6]. Further, when selecting photographs to assess PA knowledge and preferences, it is important to consider the potential bias of the items presented to children, as items may communicate which choices are healthy, and thus socially desirable. This may result in children selecting the healthy option, as they understand this as the ‘correct’ response. To address these issues, Bélanger and colleagues [30] asked teachers to take photographs of extra-curricular activities occurring within the childcare centres, whereas Parrish et al. [35] observed playground activities of children prior to developing artist-constructed drawings of the observed activities [33]. Parrish and others [35] highlighted the importance of depicting children of both genders in selected photographs, and Calfas et al. [31] identified the need to display images of children of the same age as the participants, and from varying ethnic groups. To further assist children in making a fair comparison between paired images of ‘healthy’ or preferred activities, and ‘unhealthy’ or unfavourable activities, it is important that the activities are similar in nature (both inside or outside, both solitary or group-based). Wiseman and others [6] also highlighted the importance of including pictures involving screen-time (television viewing, computer gaming) as a sedentary option for children to select, due to the increasing usage of technology by young children.

Five of the six studies that used a questionnaire that was tested for at least one type of validity and reliability. However, the low values for both scales of PA knowledge and PA preferences were concerning. Calfas and others [31] obtained non-significant results for knowledge scales by calculating the extent of improvement after a short intervention, and low reliability values for knowledge and preference scales, suggesting that these scales may not be ready for use in other studies [36]. The low reliability and validity might be a result of the instability of actual preferences in children in this age group. For example, children’s choice of activity might be situation-dependent, which means, that their choices could be influenced by what the children had done immediately before the testing [31], the weather and the novelty of the activity [6]. Thus, considering or at least noting these factors may help to reduce potential bias when administering scales of PA knowledge and preferences.

### 4.4. Observation and Activity Preferences

Due to young children’s limited verbal and motor skills, observation can be a useful technique to gain insight into children’s preferences for PA [39,46]. Observation was used in two of the reviewed studies as a basis for preparing questions, and to compare stated preferences with actual activity choices as a method for assessing the reliability of photographs as data collection tools [33,35]. While this is very participant-centric, observation represents a resource-intensive data collection technique that yields highly valid situational and contextual data. As such, observation as a technique is particularly useful to complement other data collection techniques as a means to explain participant responses [39,46].

### 4.5. Practical Implications and Future Research

The following suggestions can be made on the basis of the reviewed articles. In general, data collection techniques used to measure PA knowledge and preferences in children should include material prompts, or should incorporate structured play-based activities. This encourages children to engage in the research process. Picture sorting activities appeared to be a low-cost and effective technique to measure children’s PA knowledge and preferences. However, it was evident that more work needs to be done to ensure the validity and reliability of this technique to measure PA preferences.

Further, more consideration is needed to ensure the techniques align with the characteristics of the study population. For example, when incorporating photographs or images into data collection techniques, the activity presented needs to be age-appropriate, familiar, safe and should reflect the demographic characteristics of the study population being tested. This can be assessed through pilot-testing and observing children’s activity participation as a step in tool development. If the testing is conducted through group work, research should be monitored to prevent peer influence. Considering the specifications of assessing PA knowledge, a caretaking approach might be an effective method to elicit a child’s concept of health regarding PA, instead of decisions made on personal preferences. When assessing PA preference, it is important to consider what activities the participants engaged in immediately before the testing as this might influence the preferred activities selected during testing.

### 4.6. Limitations

This systematic review is not without limitations. The inclusion criteria required authors to sufficiently describe their data collection technique in detail, which resulted in some studies that assessed preschool children’s PA knowledge and/or preferences, being excluded. In an effort to include as many relevant studies as possible, in the instances where a study did not provide sufficient detail of the technique used, the authors contacted the authors of these papers for further information.

## 5. Conclusions

This review has examined how existing techniques to measure preschool children’s knowledge of and preference for PA align with accepted methods for the assessment of young children. Overall, a limited but disparate range of engaging techniques have been used to assess preschool children’s PA knowledge and preferences. However, limitations to these techniques are evident, with few studies testing the techniques for reliability or validity properties. This review offers educators and public health professionals a resource that will enable them to develop, select or improve upon techniques to more effectively and consistently evaluate lifestyle interventions targeting preschool children.

## Figures and Tables

**Table 1:** Summary of data collection techniques used to assess preschool children’s knowledge of and preference for physical activity.

Author, Year	Outcome of Interest	Sample	Assessment Method			Data Collection Technique				Phases	Participants Per Assessment	Time per Assessment	Quality Rating	Pilot Tested	Validity	Reliability
			Qualitative	Quantitative	Mixed Methods	Interview	SPB	Questionnaire	Observation							
Wiseman, Harris [6]	PA preferences; PA knowledge	*n* = 86 3.08–5.5 years		x			x	x		2	1	NR	good	x	*Predictive validity* (1) Knowledge scale: Calculating the extent of improvement on knowledge scores following a brief education session: t = −3.4, *p* < 0.001 (2) Preference scale: Calculating the percent agreement between stated preferences and actual choices of activities: 63% *Face validity*	*Test-retest reliability* Knowledge scale r = 0.58, *p* < 0.001 Preference scale r = 0.30 *p* < 0.01 *Scale reliability* knowledge scale α = 0.59 Preference scale α = 0.53
Lanigan [7]	PA preferences; PA knowledge	*n* = 81 3.0–5.0 years			x	x	x			2	1	NR	good		NR	*Inter-rater reliability* = 0.746. Seven cases where the difference between the raters exceeded one SD (one SD = 3.6) and were coded by a third rater.
Céspedes, Briceño [16]	PA knowledge	*n* = 1216 3.0–5.0 years		x				x		1	1	NR	good	x	*Face and content validity*	*Scale reliability* Cronbach α = 0.64
Lasky and Eichelberger [18]	PA knowledge	*n* = 75 4.0–6.0 years	x			x				4	1	NR	fair		NR	NR
Cammisa, Montrone [20]	PA preferences	*n* = 49 4.0–5.0 years	x			x	x			2	1–21	NR	good		NR	NR
Leary, Adams [23]	PA preferences	*n* = 17			x	x				1	NR	NR	poor		NR	NR
Bélanger, Waite [30]	PA preferences	*n* = 811		x			x			2	5	NR	fair	x	NR	NR
Calfas, Sallis [31]	PA preferences PA knowledge	*n* = 81 4.0–8.0 years		x			x	x		4	NR	NR	good	x	*Predictive validity* PA Knowledge scale: Calculating the extent of improvement on knowledge scores following a brief intervention: non-significant results PA Preferences scale: Calculating the percentage agreement between the stated preferences and the actual choices of activities (chance level, 56.3%)	*Test retest reliability* PA Knowledge scale: r = 0.43, *p* = 0.001 PA Preference scale: r = 0.22, *p* = 0.03
Jihene, Sonia [32]	PA preferences; PA knowledge	*n* = 577 4.0–5.0 years		x				x		2	NR	NR	fair	x	NR	NR
Lopez-Dicastillo, Grande [33]	PA preferences	*n* = 38 5.0–7.0 years	x			x			x	3	2-3	30 min	fair		NR	NR
Nguyen, Gordon [34]	PA knowledge	*n* = 53 3.88–4.96 years			x	x				2	1	10–15 min	fair	x	NR	NR
Parrish, Iverson [35]	PA preferences	*n* = 1881 4.0–9.0 years		x				x	x	1	1	10 min.	good		*Predictive validity* (1) Comparison of the Actigraph Accelerometer Activity Data and CAP Non-significant results: r = 0.299, *p* = 0.228 (2) Comparison of CAP activity preferences to observed playground activities Low activity preferences r = 0.379, *p* = 0.201 Moderate activity preferences r = 0.044, *p* = 0.886 High activity preferences r = 0.374, *p* = 0.209. *Face validity*	*Test-retest reliability* r = 0.407, *p* < 0.0001
Talbot Nix, d’Agostino Ibanez [36]	PA knowledge	*n* = 51 3.0–6.0 years		x				x		2	1	NR	fair		*NR*	*Test-retest reliability* Knowledge r = 0.37, *p* < 0.01
Darbyshire et al. (1st method) [37]	PA preferences PA knowledge	*n* = 204 4.0–12.0 years	x			x	x			3	NR (focus group)	NR	fair		NR	NR
Darbyshire, P. et al. (2nd method) [37]	PA preferences PA knowledge	*n* = 204 4.0–12.0 years	x				x			1	1	NR	fair		NR	NR

SPB* = Structured play-based activity NR = Not reported.
